# Participation in the National Cervical Screening Program Among Women Who Gave Birth in New South Wales, Australia by Place of Maternal Birth: A Data Linkage Analysis

**DOI:** 10.1111/ajo.13939

**Published:** 2025-02-13

**Authors:** Susan Yuill, Megan A. Smith, Louiza S. Velentzis, Monjura Nisha, Marion Saville, Erich V. Kliewer, Deborah Bateson, Karen Canfell

**Affiliations:** ^1^ Daffodil Centre The University of Sydney, a Joint Venture With Cancer Council NSW Sydney Australia; ^2^ School of Public Health University of Sydney Sydney Australia; ^3^ Melbourne School of Population and Global Health University of Melbourne Melbourne Victoria Australia; ^4^ The Australian Centre for the Prevention of Cervical Cancer Melbourne Victoria Australia; ^5^ Department of Cancer Control Research BC Cancer Research Institute Vancouver British Columbia Canada

**Keywords:** cancer screening tests, cervical cancer, cultural diversity, uterine

## Abstract

**Objective:**

High participation rates in the National Cervical Screening Program (NCSP) by all groups of women are required to ensure the equitable elimination of cervical cancer in Australia. In this study, we examine screening participation of overseas‐born women compared to Australian‐born women who gave birth.

**Design:**

Population‐based retrospective cohort study using linked health datasets.

**Setting and Participants:**

Women who gave birth in New South Wales between January 1, 2000 and June 30, 2017.

**Main Outcome Measures:**

Participation in the NCSP (≥ 1 cytology test) in the 3‐ and 5‐year periods prior to delivery by place of maternal birth, adjusted for multiple socio‐demographic and health characteristics.

**Results:**

Among the 1 332 669 mothers who gave birth over the study period, overall cervical screening participation in the 3‐ and 5‐year periods prior to delivery was 67.0% and 75.7%, respectively. Participation was lower for overseas‐born mothers compared to Australian‐born mothers for both the 3‐year (57.8% vs. 71.7%; adjusted odds ratio [aOR]: 0.51, 95% confidence interval [CI]: 0.50–0.51) and 5‐year (64.9% vs. 81.2%; aOR: 0.40, 95% CI: 0.40–0.40) participation periods. All groups of overseas‐born women had substantially lower screening participation compared to Australian‐born women, with the lowest relative 3‐year participation in mothers born in Southern/Central Asia (aOR: 0.30, 95% CI: 0.30–0.31), Oceania (aOR: 0.31, 95% CI: 0.30–0.32), North‐East Asia (aOR: 0.49, 95% CI: 0.48–0.50), and New Zealand (aOR: 0.49, 95% CI: 0.48–0.51).

**Conclusions:**

Overseas‐born women had around half the cervical screening participation in the period prior to birth compared to Australian‐born women. It is likely that opportunities to screen these under‐screened groups during the antenatal period, typically a time of repeated health services contact, are missed.

## 1 Introduction

Cervical cancer is predicted to be eliminated at a national level in Australia by 2028–2035, with an annual incidence of < 4 cases/100 000 women achieved [1]. Cervical screening is the cornerstone of cervical cancer elimination in the short‐ and medium‐term, because vaccination has a longer‐term effect at the population level [2]. Therefore, achieving equitable elimination across all women and people with a cervix, (hereafter referred to as women), is dependent on cervical screening program participation by all subpopulations.

The National Cervical Screening Program (NCSP) was introduced in 1991, with 2‐yearly conventional cytology recommended for sexually active, asymptomatic women aged 20–69 years. In December 2017, the recommendations changed to 5‐yearly screening with HPV 16/18 genotyping and cytology triage for non‐16/18 HPV types for women aged 25–69, and an exit test for women aged 70–74 [3]. With the introduction of HPV testing, women aged ≥ 30 who were ≥ 2 years overdue for screening had the option of self‐collection of a vaginal sample. In July 2022, eligibility was broadened to allow all screen‐eligible women to have the option of self‐collection [4].

Australia is increasingly multicultural: in 2020, 30.4% of females were born overseas [5]. People from culturally and linguistically diverse backgrounds are one of the five priority populations identified within the National Strategy for the Elimination of Cervical Cancer in Australia [6].

The antenatal period is typically a time of repeated health services contact and may be the first regular such contact for women unfamiliar with the health system due to cultural and language barriers. Pregnancy provides an opportunity to check a woman's screening status and encourage cervical screening if under‐screened. In this study, we aim to assess screening participation in overseas‐born women from all global regions at the time of delivery of their infant, compared to Australian‐born women.

## 2 Materials and Methods

### 2.1 Datasets

Record‐linkage was performed on the following datasets:

#### 2.1.1 New South Wales (NSW) Perinatal Data Collection (PDC)

The NSW PDC includes all live births and stillbirths of ≥ 20 weeks gestation or ≥ 400 g birth weight in NSW public and private hospitals and homebirths since 1994. Data includes information on the mother (demographic, medical and obstetric), labour, delivery and infant condition.

#### 2.1.2 NSW Pap Test Register (PTR)

The NSW PTR collected cervical screening test data for NSW women and contains details of cervical cytology, HPV DNA and histology tests. The PTR functioned as an “opt‐off” database; screening tests for “opt‐off” women were included but with no personal identifying details. The PTR collected data from July 1996 until it was replaced by the National Cancer Screening Register in 2018. The current analysis includes data from the cytology‐based NCSP up until June 2017.

### 2.2 Study Population

The study cohort was derived from a linked dataset including women who gave birth in NSW from January 1, 2000 until June 30, 2020. For the current analyses, women were excluded if they (i) were aged < 25 years at the time of delivery to ensure they had been eligible for screening for at least 5 years (women were eligible from age 18–20 in the cytology‐based NCSP); (ii) gave birth after June 30, 2017 (as data is only available from the NSW PTR for records up to this date); and (iii) had a missing/invalid entry for country of birth or maternal age (thus also excluding women who “opted off” the NSW PTR).

### 2.3 Statistical Analysis

We examined cervical screening participation in the 3 years (primary outcome) and 5 years (secondary outcome) prior to the infants' date of delivery. Women were defined as participating in the NCSP if they had ≥ 1 cytology test recorded during the relevant time period before the infant's birth. We used a 3‐year window for participation for our primary analyses because: (i) this is in line with previous studies [7, 8]; (ii) in the cytology‐based NCSP, women were not actively invited to attend for screening, but were sent a reminder to attend for screening if they were 3 months late (i.e., at 27 months after their last negative screening cytology test); and (iii) to allow ≥ 2 years for screening participation prior to the beginning of the current pregnancy. Women with > 1 pregnancy in the study time period were included for each pregnancy. Multiple pregnancies were included as one delivery.

Analyses were stratified by place of maternal birth (country or region) as recorded in the NSW PDC. Countries were grouped according to the major categories in the Australian Bureau of Statistics (ABS) Standard Australian Classification of Countries, 2011 (see Supporting Information, Table S1) [9]. United Kingdom/Ireland and New Zealand were reported independently, as they were the most common places of birth of immigrants throughout most of the study period.

We summarised the data using descriptive analyses. We performed logistic regression to estimate odds ratios (OR) and 95% confidence intervals (CI) for the dependent variable ‘screening participation’ (yes/no) and accounted for the clustering of > 1 delivery per mother. We adjusted for independent variables that may have impacted either screening behaviour and/or cervical precancer/cancer risk [3], including maternal age, year of delivery of infant(s), pregnancy number (> 20 weeks gestation), smoking status (yes if smoked anytime during the current pregnancy), remoteness of residence [10], and area‐level socio‐economic status (SES) of residence [11], as recorded in the NSW PDC at the time of delivery. We tested for all 2‐way interactions between place of maternal birth and other covariates: results were reported where interactions were significant (*p* < 0.05).

As time of arrival in NSW is not documented in available data, women may have been screened overseas or interstate in the specified periods prior to pregnancy. To reduce the risk of misclassification of recent arrivals who were screened in their previous place of residence, we performed a sensitivity analysis of 3‐year participation, excluding the first pregnancy recorded in the NSW PDC.

Data were analysed using STATA version 16 (STATA Corp, TX, USA).

### 2.4 Ethics Statement

Ethics approval was obtained from the NSW Population and Health Services Research Ethics Committee (approval number 2020/ETH02174).

## 3 Results

Between January 1, 2000 and June 30, 2017, there were 826 725 women who had 1 336 929 pregnancies. After the exclusion of 414 (0.03%) women with missing age and 3 846 (0.29%) women with a missing/invalid country of birth, 825 066 mothers, who had 1 332 669 deliveries, were included in the study (Table 1). Of these women, 66.1% were Australian‐born and 33.9% were overseas‐born (Asia: 16.6%, Europe: 6.3%, Africa/ Middle East: 5.3%, New Zealand: 2.3%, America: 1.9%, Oceania: 1.5%; Oceania includes women from Antarctica (0.01%), but not Australia/New Zealand). The mean age at delivery was 31.8 years (Australian‐born: 31.6, overseas‐born: 32.3). Overseas‐born women were more likely to live in a major city (93.1%) than Australian‐born women (72.5%). Overall, 9.5% of women smoked during the pregnancy, including 12.3% of Australian‐born women. Only 4.0% of overseas‐born women smoked, ranging from ≤ 2% among Asian‐born women to 17.2% for New Zealand‐born women. Area‐level SES varied widely, with 53% of North African/Middle Eastern women living in the most disadvantaged areas, compared to 6%–19% of European and Northern American women. Overall screening participation was 67.0% and 75.7% in the 3 and 5 years prior to giving birth, respectively (Table 2).

**TABLE 1:** Socio‐demographic and health characteristics of women aged ≥25 years giving birth from 1 January 2000 to 30 June 2017 in New South Wales, Australia, at the time of delivery.^a^

Place of birth^b^	Total (*N* [% of total women by delivery])	Age at birth (mean [SE])	Major city resident (*n* [%])^c^	SES area of residence (most disadvantaged/least disadvantaged [%])^d^	Pregnancy number (median [IQR]) ^e^	Smoked any time during pregnancy (*n* [%])
Australia	880 900 (66.1)	31.6 (0.00)	638 432 (72.5)	20.1/23.7	2 (1.3)	107 985 (12.3)
Overseas (all)	451 769 (33.9)	32.3 (0.01)	420 676 (93.1)	25.9/24.2	2 (1.2)	18 114 (4.0)
New Zealand	30 177 (2.3)	32.3 (0.03)	26 015 (86.2)	20.3/28.0	2 (1.3)	5196 (17.2)
Oceania and Antarctica (other)^f^	20 471 (1.5)	32.2 (0.03)	19 193 (93.8)	38.5/11.0	2 (1.4)	1661 (8.1)
United Kingdom and Ireland	46 323 (3.5)	34.0 (0.02)	40 904 (88.3)	6.4/50.5	2 (1.2)	2429 (5.2)
North‐West Europe (other)^g^	13 307 (1.0)	33.4 (0.03)	11 381 (85.5)	7.7/48.6	2 (1.2)	557 (4.2)
South and Eastern Europe	24 674 (1.9)	32.5 (0.03)	23 290 (94.4)	19.0/26.6	2 (1.2)	1494 (6.1)
North Africa and the Middle East	52 663 (4.0)	31.5 (0.02)	51 320 (97.4)	53.3/9.2	2 (1.3)	3108 (5.9)
South‐East Asia	80 259 (6.0)	32.3 (0.02)	75 778 (94.4)	37.1/15.3	2 (1.2)	1635 (2.0)
North‐East Asia	74 468 (5.6)	32.5 (0.02)	71 565 (96.1)	16.9/26.2	1 (1.2)	589 (0.8)
Southern and Central Asia	66 210 (5.0)	30.7 (0.01)	62 204 (93.9)	25.9/13.2	2 (1.2)	321 (0.5)
Northern America	11 520 (0.9)	33.2 (0.04)	9818 (85.2)	8.5/48.0	2 (1.2)	324 (2.8)
South and Central America and the Caribbean	13 451 (1.0)	33.0 (0.04)	12 584 (93.6)	17.0/29.2	2 (1.2)	365 (2.7)
Sub‐Saharan Africa	18 246 (1.4)	32.6 (0.03)	16 624 (91.1)	19.7/39.7	2 (1.3)	435 (2.4)
Total	1 332 669 (100.0)	31.8 (0.00)	1 059 108 (79.5)	22.1/23.9	2 (1.3)	126 099 (9.5)

Abbreviations: IQR: interquartile range; SE: standard error; SES: socio‐economic status.

^a^ Each pregnancy within the study period was included, but multiple births were only included as one delivery.

^b^ Place of birth was reported by individual county or grouped according to the major categories in the Australian Bureau of Statistics Standard Australian Classification of Countries, 2011 [9].

^c^ Coded according to ABS Cat. No. 1270.0.55.005 – Australian Statistical Geography Standard (ASGS): Volume 5 – Remoteness Structure, 2011 [10].

^d^ Measured using Index of Relative Socio‐economic Disadvantage, 2011 [11].

^e^ Pregnancy number >20 weeks gestation.

^f^ Oceania excluding Australia and New Zealand and Antarctica (Antarctica: 0.01% of total women)

^g^ North‐West Europe excluding United Kingdom and Ireland.

**TABLE 2:** Rates (%) and adjusted odds ratios for participation in the cervical screening program in the 3 years and 5 years prior to birth by place of maternal birth and by other socio‐demographic and health characteristics in women aged ≥ 25 years giving birth from January 1, 2000 to June 30, 2017 in New South Wales, Australia.

	3‐year participation			5‐year participation		
	*n*/*N* (%)	Unadjusted OR (95% CI)	Adjusted OR (95% CI)^a^	*n*/*N* (%)	Unadjusted OR (95% CI)	Adjusted OR (95% CI)^a^
Total	892 692/1 332 669 (67.0)			1 008 280/1 332 669 (75.7)		
Place of birth^c^						
Australia	631 795/880 900 (71.7)	1.00 (reference)	1.00 (reference)	715 161/880 900 (81.2)	1.00 (reference)	1.00 (reference)
Overseas (all)	260 897/451 769 (57.8)	***0.54 (0.53–0.54)***	***0.51 (0.50–0.51)***	293 119/451 769 (64.9)	***0.43 (0.42–0.43)***	***0.40 (0.40–0.40)***
*p*‐value		**< 0.001**	**< 0.001**		**< 0.001**	**< 0.001**
New Zealand	16 493/30 177 (54.7)	***0.48 (0.46–0.49)***	***0.49 (0.48–0.51)***	19 147/30 177 (63.4)	***0.40 (0.39–0.41)***	***0.41 (0.40–0.42)***
Oceania and Antarctica (other)^d^	8 928/20 471 (43.6)	***0.30 (0.30–0.32)***	***0.31 (0.30–0.32)***	10 862/20 471 (53.1)	***0.26 (0.25–0.27)***	***0.25 (0.24–0.26)***
United Kingdom and Ireland	33 022/46 323 (71.3)	0.98 (0.96–1.00)	***0.79 (0.77–0.81)***	35 982/46 323 (77.7)	***0.81 (0.79–0.83)***	***0.66 (0.64–0.68)***
North‐West Europe (other)^e^	8 737/13 307 (65.7)	***0.75 (0.73–0.78)***	***0.64 (0.61–0.66)***	9 488/13 307 (71.3)	***0.58 (0.55–0.60)***	***0.49 (0.47–0.51)***
South and Eastern Europe	16 095/24 674 (65.2)	***0.74 (0.72–0.76)***	***0.67 (0.65–0.69)***	17 914/24 674 (72.6)	***0.61 (0.60–0.63)***	***0.56 (0.54–0.58)***
North Africa and the Middle East	30 598/52 663 (58.1)	***0.55 (0.54–0.56)***	***0.57 (0.56–0.59)***	34 890/52 663 (66.3)	***0.45 (0.45–0.46)***	***0.45 (0.44–0.46)***
South‐East Asia	47 518/80 259 (59.2)	***0.57 (0.56–0.58)***	***0.53 (0.52–0.54)***	53 243/80 259 (66.3)	***0.46 (0.45–0.46)***	***0.42 (0.41–0.43)***
North‐East Asia	43 228/74 468 (58.0)	***0.55 (0.54–0.55)***	***0.49 (0.48–0.50)***	47 751/74 468 (64.1)	***0.41 (0.41–0.42)***	***0.38 (0.37–0.39)***
Southern and Central Asia	28 296/66 210 (42.7)	***0.29 (0.29–0.30)***	***0.30 (0.30–0.31)***	32 750/66 210 (49.5)	***0.23 (0.22–0.23)***	***0.23 (0.23–0.24)***
Northern America	7 727/11 520 (67.1)	***0.80 (0.77–0.84)***	***0.68 (0.66–0.71)***	8 393/11 520 (72.9)	***0.62 (0.59–0.65)***	***0.53 (0.51–0.56)***
South and Central America and Caribbean	8 765/13 451 (65.2)	***0.74 (0.71–0.77)***	***0.66 (0.64–0.69)***	9 879/13 451 (73.4)	***0.64 (0.61–0.67)***	***0.58 (0.56–0.61)***
Sub‐Saharan Africa	11 490/18 246 (63.0)	***0.67 (0.65–0.69)***	***0.59 (0.57–0.61)***	12 820/18 246 (70.3)	***0.55 (0.53–0.57)***	***0.47 (0.45–0.49)***
*p*‐value		**< 0.001**	**< 0.001**		**< 0.001**	**< 0.001**
Maternal age (years)						
25–29	269 234/443 598 (60.7)	***0.54 (0.53–0.54)***	***0.74 (0.73–0.74)***	308 553/443 598 (69.6)	***0.67 (0.66–0.67)***	***0.71 (0.71–0.72)***
30–34	369 388/535 680 (69.0)	1.00 (reference)	1.00 (reference)	414 581/535 680 (77.4)	1.00 (reference)	1.00 (reference)
35–39	208 711/289 596 (72.1)	***1.16 (1.15–1.17)***	***1.15 (1.14–1.16)***	233 868/289 596 (80.8)	***1.23 (1.21–1.24)***	***1.19 (1.17–1.20)***
40–44	43 043/60 502 (71.1)	***1.11 (1.09–1.13)***	***1.17 (1.15–1.19)***	48 649/60 502 (80.4)	***1.20 (1.17–1.23)***	***1.24 (1.21–1.26)***
45–49	2 242/3 063 (69.9)	1.05 (0.97–1.13)	***1.20 (1.10–1.30)***	2 442/3 063 (79.7)	***1.15 (1.05–1.26)***	***1.32 (1.20–1.45)***
50+	174/230 (75.7)	***1.40 (1.04–1.89)***	***1.50 (1.09–2.06)***	187/230 (81.3)	1.27 (0.91–1.77)	1.42 (0.99–2.04)
*p*‐value^b^		**< 0.001**	**< 0.001**		**< 0.001**	**< 0.001**
*p*‐trend^b^		**< 0.001**	**< 0.001**		**< 0.001**	**< 0.001**
Year of delivery of infant(s)						
2000	50 505/69 170 (73.0)	1.00 (reference)	1.00 (reference)	53 410/69 170 (77.2)	1.00 (reference)	1.00 (reference)
2001	48 618/67 423 (72.1)	***0.96 (0.93–0.98)***	***0.94 (0.92–0.96)***	53 609/67 423 (79.5)	***1.15 (1.12–1.17)***	***1.14 (1.11–1.17)***
2002	48 737/68 123 (71.5)	***0.93 (0.91–0.95)***	***0.90 (0.88–0.92)***	54 394/68 123 (79.8)	***1.17 (1.14–1.20)***	***1.15 (1.12–1.18)***
2003	49 208/68 943 (71.4)	***0.92 (0.90–0.94)***	***0.88 (0.86–0.90)***	54 923/68 943 (79.7)	***1.16 (1.13–1.19)***	***1.13 (1.10–1.16)***
2004	48 200/68 597 (70.3)	***0.87 (0.85–0.89)***	***0.83 (0.81–0.85)***	54 145/68 597 (78.9)	***1.11 (1.08–1.13)***	***1.08 (1.05–1.11)***
2005	50 466/72 739 (69.4)	***0.84 (0.82–0.86)***	***0.80 (0.78–0.82)***	56 971/72 739 (78.3)	***1.07 (1.04–1.09)***	***1.04 (1.01–1.07)***
2006	50 957/74 636 (68.3)	***0.80 (0.78–0.81)***	***0.76 (0.74–0.78)***	57 761/74 636 (77.4)	1.01 (0.99–1.04)	0.99 (0.96–1.01)
2007	52 450/77 878 (67.3)	***0.76 (0.75–0.78)***	***0.73 (0.71–0.74)***	59 563/77 878 (76.5)	***0.96 (0.94–0.98)***	***0.94 (0.92–0.97)***
2008	53 189/78 169 (68.0)	***0.79 (0.77–0.80)***	***0.77 (0.75–0.78)***	59 730/78 169 (76.4)	***0.96 (0.93–0.98)***	***0.96 (0.94–0.99)***
2009	54 098/78 799 (68.7)	***0.81 (0.79–0.83)***	***0.80 (0.78–0.82)***	60 593/78 799 (76.9)	0.98 (0.96–1.01)	1.01 (0.98–1.03)
2010	52 713/78 629 (67.0)	***0.75 (0.74–0.77)***	***0.74 (0.72–0.76)***	59 665/78 629 (75.9)	***0.93 (0.91–0.95)***	***0.96 (0.93–0.98)***
2011	51 839/79 921 (64.9)	***0.68 (0.67–0.70)***	***0.68 (0.66–0.70)***	59 771/79 921 (74.8)	***0.88 (0.85–0.90)***	***0.92 (0.90–0.95)***
2012	52 288/81 982 (63.8)	***0.65 (0.64–0.67)***	***0.65 (0.64–0.67)***	60 596/81 982 (73.9)	***0.84 (0.82–0.86)***	***0.89 (0.87–0.91)***
2013	50 871/80 214 (63.4)	***0.64 (0.63–0.66)***	***0.64 (0.63–0.66)***	58 252/80 214 (72.6)	***0.78 (0.76–0.80)***	***0.83 (0.81–0.85)***
2014	51 903/81 321 (63.8)	***0.65 (0.64–0.67)***	***0.66 (0.64–0.67)***	58 928/81 321 (72.5)	***0.78 (0.76–0.79)***	***0.83 (0.81–0.85)***
2015	50 772/81 124 (62.6)	***0.62 (0.60–0.63)***	***0.62 (0.60–0.63)***	58 244/81 124 (71.8)	***0.75 (0.73–0.77)***	***0.79 (0.77–0.81)***
2016	50 869/83 921 (60.6)	***0.57 (0.56–0.58)***	***0.57 (0.56–0.58)***	58 914/83 921 (70.2)	***0.70 (0.68–0.71)***	***0.74 (0.72–0.76)***
2017 (January 1–June 30)	25 009/41 080 (60.9)	***0.58 (0.56–0.59)***	***0.58 (0.56–0.59)***	28 811/41 080 (70.1)	***0.69 (0.67–0.71)***	***0.74 (0.72–0.76)***
*p*‐value^b^		**< 0.001**	**< 0.001**		**< 0.001**	**< 0.001**
*p*‐trend^b^		**< 0.001**	**< 0.001**		**< 0.001**	**< 0.001**
Pregnancy number^f^						
1	319 620/516 151 (61.9)	1.00 (reference)	1.00 (reference)	352 857/516 151 (68.4)	1.00 (reference)	1.00 (reference)
2	341 184/464 057 (73.5)	***1.71 (1.69–1.72)***	***1.67 (1.66–1.68)***	381 571/464 057 (82.2)	***2.14 (2.12–2.16)***	***2.12 (2.10–2.14)***
3	151 218/215 283 (70.2)	***1.45 (1.44–1.47)***	***1.39 (1.37–1.40)***	175 742/215 283 (81.6)	***2.06 (2.03–2.08)***	***1.96 (1.93–1.99)***
4	50 685/79 896 (63.4)	***1.07 (1.05–1.08)***	***1.08 (1.06–1.10)***	60 699/79 896 (76.0)	***1.46 (1.44–1.49)***	***1.45 (1.43–1.48)***
5	16 946/30 446 (55.7)	***0.77 (0.75–0.79)***	***0.84 (0.82–0.86)***	20 940/30 446 (68.8)	1.02 (0.99–1.05)	***1.08 (1.05–1.11)***
6+	11 842/25 096 (47.2)	***0.55 (0.53–0.57)***	***0.61 (0.59–0.63)***	15 088/25 096 (60.1)	***0.70 (0.68–0.72)***	***0.75 (0.72–0.77)***
Missing	1 197/1 740 (68.8)	***1.36 (1.22–1.50)***	***1.31 (1.18–1.46)***	1 383/1 740 (79.5)	***1.79 (1.60–2.01)***	***1.71 (1.51–1.94)***
*p*‐value^b^		**< 0.001**	**< 0.001**		**< 0.001**	**< 0.001**
*p*‐trend^b^		**< 0.001**	**< 0.001**		**< 0.001**	**< 0.001**
Smoked anytime in pregnancy						
No	823 272/1 204 463 (68.4)	1.00 (reference)	1.00 (reference)	921 867/1 204 463.00 (76.5)	1.00 (reference)	1.00 (reference)
Yes	68 039/126 099 (54.0)	***0.54 (0.54–0.55)***	***0.51 (0.50–0.51)***	84 838/126 099.00 (67.3)	***0.63 (0.62–0.64)***	***0.52 (0.51–0.53)***
Not stated/missing	1 381/2 107 (65.5)	***0.88 (0.81–0.96)***	***0.87 (0.79–0.95)***	1 575/2 107.00 (74.8)	0.91 (0.82–1.00)	***0.87 (0.78–0.97)***
*p*‐value		**< 0.001**	**< 0.001**		**< 0.001**	**< 0.001**
Remoteness of residence^g^						
Major city	713 671/1 059 108 (67.4)	1.00 (reference)	1.00 (reference)	802 198/1 059 108 (75.7)	1.00 (reference)	1.00 (reference)
Inner regional	131 360/190 001 (69.1)	***1.08 (1.07–1.10)***	***1.02 (1.01–1.04)***	150 609/190 001 (79.3)	***1.22 (1.21–1.24)***	***1.02 (1.01–1.04)***
Outer regional	40 833/60 497 (67.5)	1.01 (0.99–1.03)	1.01 (0.99–1.03)	47 022/60 497 (77.7)	***1.12 (1.09–1.14)***	0.98 (0.96–1.00)
Remote	2 846/4 673 (60.9)	***0.75 (0.71–0.81)***	***0.80 (0.75–0.86)***	3 376/4 673 (72.2)	***0.83 (0.77–0.90)***	***0.77 (0.71–0.83)***
Very remote	761/1 310 (58.1)	***0.67 (0.59–0.76)***	***0.81 (0.72–0.92)***	906/1 310 (69.2)	***0.72 (0.62–0.83)***	***0.75 (0.66–0.87)***
Other NSW/states/territories, overseas	1 957/14 549 (13.5)	***0.08 (0.07–0.08)***	***0.11 (0.10–0.13)***	2 677/14 549 (18.4)	***0.07 (0.07–0.08)***	***0.10 (0.09–0.11)***
Missing	1 264/2 531 (49.9)	***0.48 (0.45–0.52)***	***0.82 (0.74–0.90)***	1 492/2 531 (58.9)	***0.46 (0.42–0.50)***	***0.76 (0.68–0.84)***
*p*‐value^b^		**< 0.001**	**< 0.001**		**< 0.001**	**< 0.001**
*p*‐trend^b^		**< 0.001**	**0.790**		**< 0.001**	**0.002**
Area‐level socio‐economic status^h^						
Quintile 1 (most disadvantaged)	179 775/293 990 (61.2)	***0.55 (0.54–0.56)***	***0.72 (0.71–0.73)***	208 963/293 990 (71.1)	***0.57 (0.56–0.58)***	***0.72 (0.71–0.73)***
Quintile 2	179 747/278 517 (64.5)	***0.64 (0.63–0.64)***	***0.76 (0.75–0.77)***	206 207/278 517 (74.0)	***0.66 (0.65–0.67)***	***0.77 (0.76–0.78)***
Quintile 3	153 523/224 156 (68.5)	***0.76 (0.75–0.77)***	***0.84 (0.83–0.85)***	173 364/224 156 (77.3)	***0.79 (0.78–0.80)***	***0.85 (0.84–0.86)***
Quintile 4	141 252/202 374 (69.8)	***0.81 (0.80–0.82)***	***0.89 (0.87–0.90)***	157 926/202 374 (78.0)	***0.82 (0.81–0.83)***	***0.91 (0.89–0.92)***
Quintile 5 (least disadvantaged)	235 875/318 310 (74.1)	1.00 (reference)	1.00 (reference)	258 500/318 310 (81.2)	1.00 (reference)	1.00 (reference)
Missing	2 520/15 322 (16.4)	***0.07 (0.07–0.07)***	***0.53 (0.47–0.59)***	3 320/15 322 (21.7)	***0.06 (0.06–0.07)***	***0.54 (0.48–0.60)***
*p*‐value^b^		**< 0.001**	**< 0.001**		**< 0.001**	**< 0.001**
*p*‐trend^b^		**< 0.001**	**< 0.001**		**< 0.001**	**< 0.001**

*Note:* Italic/bold indicates significance. *p*‐value in bold.

Abbreviations: CI, confidence interval; OR, odds ratio.

^a^ Adjusted for age at birth of infant, year of delivery of infant, parity, smoking anytime during pregnancy, remoteness of residence, area‐level socio‐economic status.

^b^ Adjusted effects estimate and *p* values are for models with the variable included as a categorical variable; *p* trend is for models with the variable included as a continuous/ordinal variable (*p*‐trend excludes missing/invalid responses).

^c^ Place of birth was reported by individual county or grouped according to the major categories in the Australian Bureau of Statistics Standard Australian Classification of Countries, 2011 [9].

^d^ Oceania and Antarctica excluding Australia and New Zealand (includes 45 women from Antarctica).

^e^ North‐West Europe excluding the United Kingdom and Ireland.

^f^ Pregnancy number > 20 weeks gestation.

^g^ Codes are according to ABS Cat. No. 1270.0.55.005—Australian Statistical Geography Standard (ASGS): Volume 5—Remoteness Structure, 2011 [10].

^h^ Measured using the Index of Relative Socio‐economic Disadvantage, 2011 [11].

### 3.1 Three‐Year Participation

Three‐year participation was lower for overseas‐born compared to Australian‐born women (57.8% vs. 71.7%; adjusted odds ratio [aOR] 0.51, 95% CI: 0.50–0.51; *p* < 0.001) (Table 2). There was a significant difference in screening participation by place of maternal birth in both the unadjusted and adjusted analyses (*p* < 0.001). All overseas‐born groups had substantially lower participation compared to Australian‐born women, with mothers born in Southern/Central Asia (aOR: 0.30, 95% CI: 0.30–0.31), Oceania (aOR: 0.31, 95% CI: 0.30–0.32), North‐East Asia (aOR: 0.49, 95% CI: 0.48–0.50), and New Zealand (aOR 0.49, 95% CI: 0.48–0.51) having the lowest relative participation.

In addition to country of birth, 3‐year screening participation was independently associated with maternal age, year of delivery, pregnancy number, smoking, remoteness of residence and area‐level SES (all variables *p* < 0.001; Table 2). Screening participation increased with increasing maternal age at delivery (*p*‐trend < 0.001). Since the year 2000, participation trended down overall (*p*‐trend < 0.001). Participation was highest in women in their second pregnancy but decreased with increasing subsequent pregnancy number (*p*‐trend < 0.001). Compared to women residing in major cities, participation was higher in women living in inner regional areas but lower in women living in remote and very remote areas. Increasing area‐level disadvantage was associated with lower participation (*p*‐trend < 0.001).

Exclusion of the first pregnancy recorded on the NSW PDC did not improve relative 3‐year participation in women from New Zealand and Oceania (Table 3). Participation increased in women from other places of birth, and in the adjusted analysis, was no longer significantly lower in women born in the United Kingdom/Ireland.

**TABLE 3:** Sensitivity analysis: Rates (%) and adjusted odds ratios for participation in the cervical screening program in the 3 years prior to birth by maternal place of birth and by other socio‐demographic and health characteristics in women aged ≥ 25 years giving birth from January 1, 2000 to June 30, 2017 in New South Wales (NSW), Australia, *excluding the first birth* recorded in the NSW Perinatal Data Collection.

	3‐year participation		
	*n*/*N* (%)	Unadjusted OR (95% CI)	Adjusted OR (95% CI)^a^
Total	539 959/738 283 (73.1)		
Place of birth^b^			
Australia	393 681/524 302 (75.1)	1.00 (reference)	1.00 (reference)
Overseas (all)	146 278/213 981 (68.4)	***0.72 (0.71–0.73)***	***0.64 (0.63–0.65)***
*p*‐value		***p* < 0.001**	***p* < 0.001**
New Zealand	9 529/16 762 (56.8)	***0.44 (0.42–0.45)***	***0.49 (0.47–0.51)***
Oceania and Antarctica (other)^c^	5 883/12 667 (46.4)	***0.29 (0.28–0.30)***	***0.31 (0.30–0.33)***
United Kingdom and Ireland	17 331/21 495 (80.6)	***1.38 (1.33–1.43)***	0.99 (0.95–1.02)
North‐West Europe (other)^d^	4 350/5 537 (78.6)	***1.22 (1.13–1.30)***	***0.88 (0.82–0.95)***
South and Eastern Europe	8 558/11 487 (74.5)	0.97 (0.93–1.02)	***0.79 (0.76–0.83)***
North Africa and the Middle East	22 945/34 236 (67.0)	***0.67 (0.66–0.69)***	***0.77 (0.74–0.79)***
South‐East Asia	27 524/38 483 (71.5)	***0.83 (0.81–0.85)***	***0.71 (0.69–0.73)***
North‐East Asia	20 621/27 939 (73.8)	***0.93 (0.91–0.96)***	***0.69 (0.67–0.72)***
Southern and Central Asia	14 538/25 113 (57.9)	***0.46 (0.44–0.47)***	***0.41 (0.40–0.42)***
Northern America	3 932/5 028 (78.2)	***1.19 (1.11–1.28)***	***0.89 (0.82–0.96)***
South and Central America and Caribbean	4 478/5 967 (75.0)	1.00 (0.94–1.06)	***0.80 (0.75–0.86)***
Sub‐Saharan Africa	6 589/9 267 (71.1)	***0.82 (0.78–0.86)***	***0.69 (0.65–0.73)***
*p*‐value		***p* < 0.001**	***p* < 0.001**

*Note:* Italic/bold indicates significance. *p*‐value in bold.

Abbreviations: CI, confidence interval; OR, odds ratio.

^a^ Adjusted for maternal age at birth of infant, year of delivery of infant, pregnancy number > 20 weeks gestation, smoking anytime during pregnancy, remoteness of residence, area‐level socio‐economic status.

^b^ Place of birth was reported by individual county or grouped according to the major categories in the Australian Bureau of Statistics Standard Australian Classification of Countries, 2011 [9].

^c^ Oceania and Antarctica excluding Australia and New Zealand.

^d^ North‐West Europe excluding the United Kingdom and Ireland.

Interaction terms were significant for place of maternal birth and each of maternal age, year of delivery of infant(s), pregnancy number, smoking, remoteness of residence and area‐level SES (*p*‐interaction < 0.001) (Table S2). Of note, the higher odds of participation among those residing in inner regional areas compared to their compatriots residing in a major city was only observed for women born in Australia, New Zealand and Oceania. Participation was lower in immigrant women from all regions who smoked during their pregnancy except for Southern/Central Asian women.

### 3.2 Five‐Year Participation

Five‐year participation was lower for overseas‐born compared to Australian‐born women (64.9% vs. 81.2%; aOR: 0.40, 95% CI: 0.40–0.40, *p* < 0.001; Table 2). (See Tables 2,S3 for further results).

## 4 Discussion

All groups of overseas‐born women giving birth in NSW between 2000 and 2017 had significantly lower cervical screening participation in the 3 and 5 years prior to delivery compared to Australian women. The proportion screened and relative participation varied by place of maternal birth. Women from Southern/Central Asia and Oceania had particularly low relative participation, with less than a third of the odds of being screened within 3 years prior to delivery, while New Zealand and North‐East Asian women had less than half the odds of being screened.

A previous meta‐analysis found reduced cervical screening participation in some immigrant groups in Australia, with consistently lower screening rates in South Asian women [12]. An included study of Asian and Middle Eastern women who gave birth in NSW in 2000 found lower 3‐year screening participation in both groups compared to Australian‐born women, adjusted for age, parity, smoking and SES [7]. We found lower relative participation in immigrant women from all geographical regions. We also accounted for the remoteness of residence, which has been shown to impact screening participation [13].

In a recent analysis of women aged ≥ 45 years in NSW, 3‐year cervical screening participation between 2010 and 2012 was lower in women born in New Zealand, Oceania, the Middle East/North Africa, and most Asian countries/regions, adjusted for socio‐demographic, lifestyle and health factors [8]. Compared to the present study, participation and relative participation were higher in all groups of overseas‐born women. This may partly be due to the younger age of women in the current study (94.9% were aged < 40), as screening participation increased with increasing maternal age. The current study included all women giving birth in NSW between 2000 and 2017, whereas the 45 and Up Study population included a random sample from the general NSW resident population aged ≥ 45 who spoke English and were screened between 2008 and 2012.

An important finding of concern was that some groups of overseas‐born women in their second or later recorded pregnancy still had substantially lower screening within the previous 3 years. The antenatal period provides an opportunity to offer cervical screening, as pregnancy may be one of the few times women interact with the health system, particularly women who face language and cultural barriers.

Also concerning was decreasing screening participation over the study period in all groups of women, Australian and overseas‐born, which is consistent with overall trends in NSW and Australia [14, 15]. Compared to Australian‐born women who gave birth in the year 2000, the odds of being screened in the 3 years prior to giving birth in 2017 was around half in Australian‐born women and less than a fifth in women born in Oceania and Southern/Central Asia. Screening participation decreased with increasing area‐level disadvantage, reflecting national patterns [16], but was lowest in overseas‐born women residing in areas of highest disadvantage. Participation was highest in women from inner regional areas and lowest in women living in remote/very remote areas, also reflecting national patterns [16].

Our study was limited as women screened outside NSW in the specified windows before delivery were misclassified as non‐participants. The proportion of the overseas‐born Australian population increases from the 20–24 year age group [13] and thus the childbearing years are a common time for women to immigrate to Australia. This may also explain the lower relative 5‐year than 3‐year participation rates in all immigrant groups, as a greater proportion of immigrant women may not have been living in NSW for the 5 years prior to delivery. To address this, we examined 3‐year participation rates of the second or subsequent recorded pregnancies. While this improved relative screening rates in most groups, there was no change in participation in some groups with the lowest participation, that is, Oceania and New Zealand. Recommendations during the cytology‐based NCSP were to commence screening 1–2 years after becoming sexually active. Women influenced by cultural norms may thus not have been screened prior to their first pregnancy.

A further limitation of this study is that cervical screening involved 2‐yearly cytology throughout the study period, rather than the currently recommended 5‐yearly HPV testing. A comparable analysis is not yet possible with data from the HPV‐based NCSP to assess the impact of the change to 5‐yearly HPV testing (initially with limited eligibility for self‐collection) in December 2017, and the broadening of the option of self‐collection to all screen‐eligible women in July 2022. While country of birth is requested for the National Cancer Screening Register (NCSR), it is not completed for 85% of women [17]. Looking at other priority groups with lower screening participation in this study, at the end of 2022, participation in the HPV‐based screening program remained lower in women residing in very remote areas (69% were up to date with screening compared to 78% in women residing in major cities), and varied by SES (coverage was highest in women living in the highest SES areas (quintile of least disadvantage), and was lowest in women in the middle quintile, but was above 70% in all quintiles) [18]. Similarly, there are no data available for self‐collection of an HPV sample by place of birth. However, since the change to allow all screen‐eligible women the option of a self‐collected sample in July 2022, the proportion of screening tests that were self‐collected has consistently increased, from less than 5% in June 2022 to 43% in October 2024 [19]. Some women undergoing self‐collection are those who have routinely participated in the screening program and previously had a clinician‐collected sample (19% during 2023) [18]. However, the proportion of tests that are self‐collected has particularly increased among women who were previously never screened (41% of screening tests in this group in April–June 2024, compared to 34% nationally) and under‐screened (50% of tests in April–June 2024), and the greatest increase was seen in women living in the most disadvantaged areas and in those living remotely [20].

Furthermore, it has been shown that a single measure such as country of birth does not fully capture cultural, ethnic and linguistic diversity, and does not take into account time lived in Australia or English fluency [21]. Future linkage of the NCSR to Census data (a rich source of cultural, ethnic and linguistic data) through the Person Level Integrated Data Asset (PLIDA) [21, 22], would allow more in‐depth exploration of ethnicity/ancestry and may, for example, identify under‐screened subgroups of New Zealand‐born women living in Australia. However, until these data become available, this study provides important evidence to inform strategies for addressing under‐screening in immigrant women.

Our study has several important strengths. We used routinely collected health data from statewide datasets rather than self‐reported data. Our dataset was large (with 1 336 732 pregnancies in 825 066 women), included all NSW births between 2000 and 2017, and contained women born in all geographical regions. We adjusted for multiple factors that have been associated with screening participation and/or cervical precancer/cancer in Australia [3].

Cervical screening in pregnancy is safe, provided the correct sampling equipment is used. The cervix is hyperaemic, and post‐cervical‐sampling bleeding may distress some pregnant women. Recommendations for screening during pregnancy with cytology‐based screening programs varied. In the United Kingdom, cytology screening is not recommended during pregnancy [23, 24]. While there were no specific recommendations in previous Australian guidelines for cytology‐based screening [25, 26], some antenatal clinics recommended screening only in women ≤ 20 weeks gestation due for screening. Those not screened were recommended to have a Pap smear postnatally after 6 weeks, preferably at 3 months, as cytology interpretation can be difficult in the immediate postpartum period [3]. A speculum examination in the postnatal period may be associated with discomfort, especially if breastfeeding. However, HPV testing can be performed any time during pregnancy or postpartum, and since December 2017, Australian cervical screening guidelines have explicitly stated that routine antenatal and postpartum care should include a review of the woman's cervical screening history, and those due or overdue for screening should be screened [3]. The universal option of HPV‐sample self‐collection since July 2022 [4] allows testing to be performed without a speculum examination, which may be more acceptable to women during pregnancy and postnatally. Postpartum follow‐up, if required, should optimally be performed at 3 months to reduce the risk of difficulties interpreting reflex cytology [3].

Our study of women giving birth found lower screening participation among immigrant women from all geographical regions. The antenatal period provides opportunities to offer cervical screening. HPV testing with the option of self‐collection may be more acceptable to overseas‐born and other groups of under‐screened women. As data become available, future studies and ongoing surveillance should examine the impact of the change to 5‐yearly HPV testing and the universal option of self‐collection on cervical screening participation in immigrant women and women giving birth.

## Conflicts of Interest

M.S. receives contract funding to her institution from the Commonwealth Department of Health (Australia), the Ministry of Health (New Zealand), the National Screening Service (Ireland), and the Department of Social Care (UK) for projects related to cervical cancer control. K.C., D.B., and M.A.S. are co‐PIs on the implementation program Elimination of Cervical Cancer in the Western Pacific, which has received support from the Minderoo Foundation and equipment donations from Cepheid Inc. K.C. is a co‐PI of an investigator‐initiated trial of cervical screening, *Compass*, run by the Australian Centre for Prevention of Cervical Cancer (ACPCC), which is a government‐funded not‐for‐profit charity; the ACPCC has received equipment and a funding contribution from Roche Molecular Diagnostics and operational support from the Australian Government. M.A.S is a co‐PI on the *Compass* trial, for which her organisation, ACPCC, has received kits and partial funding from Roche. VCS Pathology has also received free test kits from Roche, Seegene, Cepheid, Becton Dickinson, Abbott, AusDiagnostics and Atila Biosystems for research purposes. K.C. receives contract funding from the Commonwealth Department of Health, Australia, to her institution for work to monitor the safety of the National Cervical Screening Program. K.C. also receives support for a range of other Australian and international government projects, including support from philanthropic organisations, WHO, and government agencies related to cervical cancer control. The remaining authors (S.Y., L.S.V.) declare no conflicts of interest.

## Supporting information


Data S1.
