# Supplementary material for: Participation in the National Cervical Screening Program Among Women Who Gave Birth in New South Wales, Australia by Place of Maternal Birth: A Data Linkage Analysis
Source: Aust N Z J Obstet Gynaecol. 2025 Feb 13;65(4):518–29. doi: 10.1111/ajo.13939 (PMC12668881; doi:10.1111/ajo.13939)
Supplement: Supplementary file 1 — Data S1. [file AJO-65-518-s001.docx]

# Supplement

Table S1: Thirteen categories of place of maternal birth.

| **Place of birth** | **Countries and minor groups included** |
| --- | --- |
| **Australia** | Australia |
| **New Zealand** | New Zealand |
| **Oceania and Antarctica (other)** | Melanesia, Micronesia, Polynesia (excludes Hawaii), Antarctica, excluding Australia and New Zealand |
| **United Kingdom and Ireland** | United Kingdom, Ireland |
| **North-West Europe (other)** | Western Europe (includes Germany), Northern Europe, excluding United Kingdom and Ireland |
| **South and Eastern Europe** | Southern Europe (includes Italy), South Eastern Europe, Eastern Europe |
| **North Africa and the Middle East** | North Africa, Middle East |
| **South-East Asia** | Mainland South-East Asia (includes Vietnam), Maritime South-East Asia (includes Philippines and Malaysia) |
| **North-East Asia** | Chinese Asia (includes China), Japan and the Koreas |
| **Southern and Central Asia** | Southern Asia (includes India) |
| **Northern America** | Northern America (includes United States of America and Canada) |
| **South America, Central America and the Caribbean** | South America, Central America and the Caribbean |
| **Sub-Saharan Africa** | Central and West Africa, Southern and Eastern Africa (includes South Africa) |

Countries are grouped in categories according to major groups as defined by the Australian Bureau of Statistics (ABS) Standard Australian Classification of Countries, 2011^1^ with the exception of Australia, and the United Kingdom/Ireland and New Zealand, the most common places of birth of immigrants in 2011.^2^

Table S2: Rates of participation~ (%) and adjusted odds ratios for participation in the cervical screening program in the 3-years prior to birth by place of maternal birth by categories of socio-demographic and health characteristics in women aged ≥25 years giving birth from 1 January 2000 to 30 June 2017 in New South Wales, Australia.

| Place of birth~ | % | Adjusted OR*  (95%CI) | % | Adjusted OR*  (95%CI) | % | Adjusted OR*  (95%CI) | % | Adjusted OR*  (95%CI) | % | Adjusted OR*  (95%CI) | % | Adjusted OR*  (95%CI) |
| --- | --- | --- | --- | --- | --- | --- | --- | --- | --- | --- | --- | --- |
|  | **Maternal age** | | | | | | | | | |  |  |
|  | **25-29 years** | | **30-34 years** | | **35-39 years** | | **40-44 years** | | **45+ years** | |  |  |
| Australia | 66.5 | ***0.76 (0.75-0.77)*** | 73.9 | 1.00 (reference) | 75.8 | ***1.10 (1.09-1.12)*** | 74.6 | ***1.10 (1.07-1.13)*** | 73.5 | 1.09 (0.98-1.21) |  |  |
| New Zealand | 43.1 | ***0.31 (0.29-0.32)*** | 56.4 | ***0.50 (0.48-0.52)*** | 63.3 | ***0.65 (0.62-0.69)*** | 65.8 | ***0.75 (0.67-0.83)*** | 57.6 | ***0.60 (0.38-0.95)*** |  |  |
| Oceania and Antarctica (other)^a^ | 37.5 | ***0.21 (0.20-0.23)*** | 46.6 | ***0.32 (0.30-0.34)*** | 46.6 | ***0.35 (0.33-0.37)*** | 45.2 | ***0.37 (0.33-0.42)*** | 51.3 | ***0.54 (0.33-0.86)*** |  |  |
| United Kingdom and Ireland | 60.5 | ***0.55 (0.52-0.58)*** | 69.9 | ***0.76 (0.73-0.79)*** | 75.9 | 0.98 (0.94-1.01) | 75.4 | 0.99 (0.92-1.07) | 75.5 | 1.07 (0.78-1.48) |  |  |
| North-West Europe (other)^b^ | 54.0 | ***0.42 (0.39-0.46)*** | 65.4 | ***0.63 (0.60-0.67)*** | 71.9 | ***0.80 (0.75-0.86)*** | 69.4 | ***0.72 (0.62-0.84)*** | 63.0 | 0.67 (0.35-1.29) |  |  |
| South and Eastern Europe | 57.9 | ***0.49 (0.47-0.52)*** | 66.2 | ***0.67 (0.64-0.70)*** | 70.5 | ***0.79 (0.75-0.84)*** | 68.5 | ***0.75 (0.67-0.84)*** | 70.7 | 0.89 (0.58-1.37) |  |  |
| North Africa and the Middle East | 52.3 | ***0.40 (0.39-0.41)*** | 60.2 | ***0.57 (0.55-0.59)*** | 63.9 | ***0.72 (0.69-0.75)*** | 63.9 | ***0.80 (0.73-0.87)*** | 61.7 | 0.76 (0.55-1.06) |  |  |
| South-East Asia | 50.8 | ***0.37 (0.36-0.39)*** | 60.9 | ***0.54 (0.52-0.55)*** | 65.0 | ***0.63 (0.61-0.65)*** | 65.3 | ***0.66 (0.61-0.70)*** | 65.7 | ***0.74 (0.56-0.97)*** |  |  |
| North-East Asia | 47.3 | ***0.34 (0.33-0.35)*** | 59.6 | ***0.50 (0.49-0.51)*** | 65.1 | ***0.58 (0.56-0.60)*** | 65.8 | ***0.60 (0.56-0.64)*** | 71.1 | 0.82 (0.60-1.12) |  |  |
| Southern and Central Asia | 34.7 | ***0.21 (0.20-0.21)*** | 46.5 | ***0.31 (0.30-0.31)*** | 54.1 | ***0.39 (0.38-0.41)*** | 56.1 | ***0.45 (0.40-0.50)*** | 50.7 | ***0.45 (0.26-0.76)*** |  |  |
| Northern America | 55.4 | ***0.45 (0.41-0.49)*** | 67.1 | ***0.68 (0.64-0.73)*** | 73.0 | ***0.84 (0.78-0.91)*** | 75.7 | 0.97 (0.82-1.15) | 68.3 | 0.69 (0.39-1.20) |  |  |
| South and Central America and Caribbean | 57.6 | ***0.49 (0.45-0.53)*** | 64.2 | ***0.64 (0.60-0.67)*** | 71.0 | ***0.82 (0.76-0.88)*** | 70.3 | ***0.79 (0.67-0.92)*** | 69.6 | 0.84 (0.48-1.48) |  |  |
| Sub-Saharan Africa | 54.5 | ***0.43 (0.40-0.45)*** | 65.1 | ***0.61 (0.58-0.64)*** | 67.6 | ***0.67 (0.63-0.71)*** | 64.6 | ***0.64 (0.56-0.73)*** | 65.5 | 0.71 (0.42-1.20) |  |  |
| p (interaction) |  |  |  |  |  | **p<0.001** |  |  |  |  |  |  |
|  | **Year of delivery of infant(s)** | | | | | | | | | |  |  |
|  | **2000** | | **2005** | | **2010** | | **2015** | | **2017 (to June 30)** | |  |  |
| Australia | 76.6 | 1.00 (reference) | 73.3 | ***0.79 (0.77-0.81)*** | 72.0 | ***0.72 (0.70-0.74)*** | 67.8 | ***0.58 (0.56-0.60)*** | 66.2 | ***0.54 (0.52-0.56)*** |  |  |
| New Zealand | 61.1 | ***0.48 (0.43-0.54)*** | 55.1 | ***0.37 (0.33-0.41)*** | 57.3 | ***0.39 (0.35-0.43)*** | 52.9 | ***0.32 (0.29-0.36)*** | 49.7 | ***0.28 (0.24-0.32)*** |  |  |
| Oceania and Antarctica (other)^a^ | 46.4 | ***0.26 (0.23-0.29)*** | 45.7 | ***0.24 (0.21-0.27)*** | 44.3 | ***0.23 (0.21-0.26)*** | 39.9 | ***0.19 (0.17-0.22)*** | 39.2 | ***0.18 (0.15-0.23)*** |  |  |
| United Kingdom and Ireland | 75.0 | ***0.75 (0.69-0.83)*** | 70.8 | ***0.55 (0.51-0.61)*** | 71.8 | ***0.58 (0.53-0.63)*** | 71.4 | ***0.56 (0.51-0.61)*** | 71.8 | ***0.56 (0.50-0.64)*** |  |  |
| North-West Europe (other)^b^ | 72.1 | ***0.64 (0.53-0.76)*** | 63.9 | ***0.42 (0.36-0.49)*** | 64.8 | ***0.43 (0.37-0.49)*** | 63.6 | ***0.40 (0.34-0.46)*** | 61.9 | ***0.37 (0.31-0.45)*** |  |  |
| South and Eastern Europe | 68.3 | ***0.58 (0.51-0.65)*** | 65.8 | ***0.50 (0.44-0.57)*** | 67.7 | ***0.53 (0.47-0.59)*** | 61.1 | ***0.38 (0.34-0.42)*** | 60.8 | ***0.38 (0.33-0.43)*** |  |  |
| North Africa and the Middle East | 64.9 | ***0.57 (0.53-0.62)*** | 60.7 | ***0.45 (0.42-0.49)*** | 57.6 | ***0.40 (0.37-0.43)*** | 53.0 | ***0.32 (0.30-0.35)*** | 52.2 | ***0.30 (0.27-0.33)*** |  |  |
| South-East Asia | 64.9 | ***0.50 (0.47-0.54)*** | 60.5 | ***0.40 (0.38-0.43)*** | 58.4 | ***0.37 (0.34-0.39)*** | 55.7 | ***0.32 (0.30-0.34)*** | 53.6 | ***0.29 (0.27-0.32)*** |  |  |
| North-East Asia | 61.0 | ***0.38 (0.35-0.40)*** | 60.3 | ***0.37 (0.34-0.40)*** | 57.9 | ***0.34 (0.32-0.36)*** | 57.1 | ***0.32 (0.30-0.34)*** | 56.7 | ***0.31 (0.29-0.33)*** |  |  |
| Southern and Central Asia | 55.1 | ***0.31 (0.28-0.35)*** | 45.5 | ***0.22 (0.20-0.24)*** | 42.3 | ***0.20 (0.19-0.22)*** | 40.7 | ***0.18 (0.17-0.19)*** | 41.8 | ***0.19 (0.17-0.20)*** |  |  |
| Northern America | 72.9 | ***0.64 (0.53-0.79)*** | 66.7 | ***0.47 (0.39-0.57)*** | 68.3 | ***0.52 (0.44-0.61)*** | 64.7 | ***0.43 (0.37-0.51)*** | 64.3 | ***0.41 (0.33-0.51)*** |  |  |
| South and Central America and Caribbean | 73.6 | ***0.72 (0.60-0.87)*** | 65.9 | ***0.48 (0.41-0.57)*** | 61.5 | ***0.40 (0.34-0.46)*** | 63.6 | ***0.42 (0.37-0.48)*** | 60.3 | ***0.36 (0.30-0.43)*** |  |  |
| Sub-Saharan Africa | 68.9 | ***0.53 (0.44-0.63)*** | 66.5 | ***0.49 (0.43-0.57)*** | 66.4 | ***0.48 (0.43-0.55)*** | 60.7 | ***0.37 (0.33-0.42)*** | 57.6 | ***0.33 (0.28-0.39)*** |  |  |
| p (interaction) | **p<0.001** | | | | | | | | | |  |  |
|  | **Pregnancy number^Ɨ^** | | | | | | | | | | | |
|  | **1** | | **2** | | **3** | | **4** | | **5** | | **6+** | |
| Australia | 68.8 | 1.00 (reference) | 77.8 | ***1.59 (1.57-1.60)*** | 72.8 | ***1.26 (1.24-1.28)*** | 65.3 | ***0.95 (0.93-0.96)*** | 57.0 | ***0.71 (0.69-0.74)*** | 48.1 | ***0.51 (0.49-0.53)*** |
| New Zealand | 58.0 | ***0.61 (0.58-0.63)*** | 62.8 | ***0.77 (0.74-0.81)*** | 51.8 | ***0.53 (0.50-0.56)*** | 41.8 | ***0.38 (0.35-0.41)*** | 31.5 | ***0.20 (0.18-0.23)*** | 29.8 | ***0.18 (0.16-0.20)*** |
| Oceania and Antarctica (other)^a^ | 42.9 | ***0.34 (0.33-0.36)*** | 53.6 | ***0.51 (0.49-0.54)*** | 44.4 | ***0.36 (0.33-0.38)*** | 36.5 | ***0.25 (0.23-0.28)*** | 34.0 | ***0.28 (0.25-0.31)*** | 30.7 | ***0.24 (0.21-0.27)*** |
| United Kingdom and Ireland | 66.8 | ***0.77 (0.75-0.79)*** | 77.4 | ***1.27 (1.22-1.32)*** | 72.8 | ***0.99 (0.93-1.05)*** | 67.6 | ***0.78 (0.70-0.87)*** | 67.6 | ***0.82 (0.55-1.22)*** | 62.1 | ***0.64 (0.35-1.17)*** |
| North-West Europe (other)^b^ | 60.1 | ***0.60 (0.57-0.64)*** | 72.6 | ***1.01 (0.95-1.08)*** | 68.5 | ***0.80 (0.72-0.90)*** | 64.3 | ***0.69 (0.56-0.86)*** | 63.6 | ***0.69 (0.57-0.83)*** | 52.5 | ***0.45 (0.35-0.59)*** |
| South and Eastern Europe | 58.8 | ***0.62 (0.60-0.65)*** | 71.3 | 1.05 (1.00-1.10) | 70.5 | ***0.98 (0.90-1.05)*** | 67.2 | ***0.83 (0.72-0.96)*** | 60.5 | ***0.63 (0.48-0.82)*** | 53.1 | ***0.45 (0.31-0.67)*** |
| North Africa and the Middle East | 40.9 | ***0.34 (0.32-0.35)*** | 63.5 | ***0.84 (0.81-0.87)*** | 65.8 | ***0.92 (0.89-0.96)*** | 65.5 | ***0.88 (0.84-0.93)*** | 64.6 | ***0.83 (0.77-0.90)*** | 58.3 | ***0.61 (0.56-0.67)*** |
| South-East Asia | 49.2 | ***0.44 (0.43-0.45)*** | 67.9 | ***0.92 (0.89-0.94)*** | 66.8 | ***0.84 (0.81-0.88)*** | 62.6 | ***0.69 (0.64-0.74)*** | 56.5 | ***0.54 (0.47-0.61)*** | 50.8 | ***0.43 (0.36-0.52)*** |
| North-East Asia | 49.9 | ***0.43 (0.43-0.44)*** | 67.0 | ***0.81 (0.79-0.84)*** | 68.4 | ***0.84 (0.79-0.89)*** | 67.1 | ***0.79 (0.69-0.90)*** | 65.8 | ***0.75 (0.57-0.99)*** | 62.0 | ***0.62 (0.39-1.00)*** |
| Southern and Central Asia | 33.5 | ***0.26 (0.25-0.27)*** | 50.1 | ***0.47 (0.45-0.48)*** | 54.7 | ***0.54 (0.51-0.57)*** | 55.9 | ***0.55 (0.50-0.61)*** | 53.1 | ***0.48 (0.41-0.57)*** | 48.5 | ***0.39 (0.31-0.48)*** |
| Northern America | 63.0 | ***0.69 (0.65-0.73)*** | 72.8 | 1.04 (0.96-1.11) | 68.8 | ***0.83 (0.74-0.93)*** | 62.8 | ***0.66 (0.54-0.81)*** | 60.0 | ***0.60 (0.41-0.88)*** | 58.8 | ***0.55 (0.36-0.84)*** |
| South and Central America and Caribbean | 59.1 | ***0.62 (0.59-0.65)*** | 72.1 | 1.06 (0.99-1.13) | 69.3 | 0.91 (0.82-1.01) | 67.4 | 0.83 (0.69-1.01) | 56.4 | ***0.54 (0.38-0.76)*** | 61.0 | ***0.60 (0.38-0.95)*** |
| Sub-Saharan Africa | 59.3 | ***0.61 (0.58-0.64)*** | 68.2 | ***0.87 (0.82-0.91***) | 64.0 | ***0.72 (0.67-0.78)*** | 60.4 | ***0.63 (0.55-0.71)*** | 53.3 | ***0.49 (0.40-0.60)*** | 51.1 | ***0.44 (0.34-0.56)*** |
| p (interaction) | **<0.001** | | | | | | | | | | | |
|  | **Smoked anytime in pregnancy** | | | |  |  |  |  |  |  |  |  |
|  | **No smoking** | | **Yes smoking** | |  |  |  |  |  |  |  |  |
| Australia | 74.1 | 1.00 (reference) | 55.1 | ***0.48 (0.48-0.49)*** |  |  |  |  |  |  |  |  |
| New Zealand | 58.2 | ***0.49 (0.48-0.51)*** | 38.0 | ***0.25 (0.23-0.26)*** |  |  |  |  |  |  |  |  |
| Oceania and Antarctica (other)^a^ | 44.9 | ***0.30 (0.29-0.31)*** | 29.0 | ***0.17 (0.15-0.19)*** |  |  |  |  |  |  |  |  |
| United Kingdom and Ireland | 72.1 | ***0.79 (0.77-0.80)*** | 56.0 | ***0.41 (0.38-0.45)*** |  |  |  |  |  |  |  |  |
| North-West Europe (other)^b^ | 66.1 | ***0.63 (0.60-0.65)*** | 54.2 | ***0.40 (0.33-0.48)*** |  |  |  |  |  |  |  |  |
| South and Eastern Europe | 65.7 | ***0.65 (0.63-0.67)*** | 57.5 | ***0.46 (0.42-0.52)*** |  |  |  |  |  |  |  |  |
| North Africa and the Middle East | 58.4 | ***0.55 (0.54-0.56)*** | 53.8 | ***0.46 (0.42-0.49)*** |  |  |  |  |  |  |  |  |
| South-East Asia | 59.4 | ***0.52 (0.51-0.53)*** | 51.4 | ***0.41 (0.37-0.46)*** |  |  |  |  |  |  |  |  |
| North-East Asia | 58.1 | ***0.49 (0.48-0.49)*** | 47.7 | ***0.35 (0.29-0.41)*** |  |  |  |  |  |  |  |  |
| Southern and Central Asia | 42.7 | ***0.30 (0.29-0.30)*** | 52.3 | ***0.43 (0.34-0.55)*** |  |  |  |  |  |  |  |  |
| Northern America | 67.5 | ***0.68 (0.65-0.71)*** | 51.5 | ***0.37 (0.29-0.47)*** |  |  |  |  |  |  |  |  |
| South and Central America and Caribbean | 65.4 | ***0.65 (0.63-0.68)*** | 58.1 | ***0.50 (0.40-0.63)*** |  |  |  |  |  |  |  |  |
| Sub-Saharan Africa | 63.2 | ***0.58 (0.56-0.60)*** | 54.7 | ***0.41 (0.34-0.51)*** |  |  |  |  |  |  |  |  |
| p (interaction) | **p<0.001** | | | |  |  |  |  |  |  |  |  |
|  | **Remoteness of residence**^#^ | | | | | | | |  |  |  |  |
|  | **Major city** | | **Inner regional** | | **Outer regional** | | **Remote/very remote** | |  |  |  |  |
| Australia | 73.2 | 1.00 (reference) | 70.5 | ***1.05 (1.04-1.07)*** | 69.0 | ***1.05 (1.03-1.07)*** | 61.1 | ***0.81 (0.76-0.86)*** |  |  |  |  |
| New Zealand | 55.7 | ***0.49 (0.47-0.50)*** | 60.1 | ***0.63 (0.57-0.68)*** | 53.7 | ***0.52 (0.44-0.62)*** | 56.4 | 0.60 (0.34-1.05) |  |  |  |  |
| Oceania and Antarctica (other)^a^ | 44.1 | ***0.31 (0.30-0.32)*** | 50.0 | ***0.38 (0.32-0.45)*** | 34.7 | ***0.24 (0.19-0.31)*** | 30.8 | ***0.19 (0.07-0.48)*** |  |  |  |  |
| United Kingdom and Ireland | 72.8 | ***0.81 (0.79-0.83)*** | 66.8 | ***0.71 (0.66-0.76)*** | 63.0 | ***0.64 (0.54-0.76)*** | 58.1 | 0.60 (0.34-1.06) |  |  |  |  |
| North-West Europe (other)^b^ | 67.3 | ***0.64 (0.62-0.67)*** | 61.8 | ***0.58 (0.52-0.66)*** | 61.7 | ***0.63 (0.49-0.81)*** | 66.7 | 0.93 (0.29-2.93) |  |  |  |  |
| South and Eastern Europe | 66.2 | ***0.68 (0.66-0.70)*** | 58.8 | ***0.53 (0.46-0.61)*** | 51.8 | ***0.41 (0.30-0.57)*** | ǂ | 0.59 (0.15-2.32) |  |  |  |  |
| North Africa and the Middle East | 58.5 | ***0.58 (0.57-0.59)*** | 50.6 | ***0.43 (0.37-0.50)*** | 43.5 | ***0.31 (0.21-0.45)*** | ǂ | 0.32 (0.07-1.36) |  |  |  |  |
| South-East Asia | 60.3 | ***0.54 (0.53-0.55)*** | 50.7 | ***0.39 (0.36-0.42)*** | 44.5 | ***0.31 (0.26-0.37)*** | 48.1 | ***0.37 (0.21-0.65)*** |  |  |  |  |
| North-East Asia | 58.9 | ***0.49 (0.49-0.50)*** | 51.2 | ***0.39 (0.35-0.43)*** | 45.9 | ***0.33 (0.26-0.42)*** | 52.4 | 0.46 (0.20-1.05) |  |  |  |  |
| Southern and Central Asia | 43.8 | ***0.31 (0.30-0.31)*** | 35.6 | ***0.24 (0.21-0.26)*** | 26.5 | ***0.15 (0.13-0.18)*** | 20.8 | ***0.12 (0.06-0.25)*** |  |  |  |  |
| Northern America | 69.0 | ***0.70 (0.67-0.73)*** | 61.7 | ***0.60 (0.52-0.68)*** | 64.6 | ***0.69 (0.53-0.89)*** | 52.6 | 0.42 (0.16-1.12) |  |  |  |  |
| South and Central America and Caribbean | 66.6 | ***0.68 (0.65-0.70)*** | 56.4 | ***0.48 (0.39-0.57)*** | 50.6 | ***0.39 (0.24-0.64)*** | ǂ | 0.50 (0.13-1.94) |  |  |  |  |
| Sub-Saharan Africa | 64.1 | ***0.60 (0.58-0.62)*** | 58.0 | ***0.53 (0.46-0.60)*** | 57.9 | ***0.53 (0.39-0.73)*** | 40.0 | ***0.24 (0.09-0.61)*** |  |  |  |  |
| p (interaction) | **p<0.001** | | | | | | | |  |  |  |  |
|  | **Area-level socio-economic status (Quintile)**≈ | | | | | | | | | |  |  |
|  | **1 (most disadvantaged)** | | **2** | | **3** | | **4** | | **5 (least disadvantaged)** | |  |  |
| Australia | 65.2 | ***0.66 (0.65-0.67)*** | 69.7 | ***0.76 (0.75-0.77)*** | 73.0 | ***0.85 (0.83-0.86)*** | 75.1 | ***0.90 (0.89-0.92)*** | 78.2 | 1.00 (reference) |  |  |
| New Zealand | 39.5 | ***0.25 (0.23-0.27)*** | 49.3 | ***0.33 (0.32-0.35)*** | 55.5 | ***0.41 (0.38-0.43)*** | 61.3 | ***0.49 (0.45-0.52)*** | 69.9 | ***0.65 (0.61-0.68)*** |  |  |
| Oceania and Antarctica (other)^a^ | 36.2 | ***0.18 (0.17-0.19)*** | 42.7 | ***0.22 (0.21-0.24)*** | 45.8 | ***0.25 (0.23-0.27)*** | 53.4 | ***0.33 (0.30-0.36)*** | 63.6 | ***0.47 (0.43-0.52)*** |  |  |
| United Kingdom and Ireland | 64.9 | ***0.55 (0.51-0.60)*** | 67.1 | ***0.58 (0.55-0.62)*** | 71.7 | ***0.70 (0.66-0.74)*** | 72.8 | ***0.73 (0.69-0.77)*** | 74.0 | ***0.76 (0.73-0.78)*** |  |  |
| North-West Europe (other)^b^ | 61.1 | ***0.46 (0.40-0.53)*** | 63.9 | ***0.52 (0.46-0.57)*** | 66.2 | ***0.56 (0.50-0.62)*** | 67.6 | ***0.60 (0.55-0.66)*** | 68.0 | ***0.58 (0.55-0.61)*** |  |  |
| South and Eastern Europe | 63.0 | ***0.51 (0.47-0.54)*** | 63.8 | ***0.52 (0.49-0.56)*** | 66.1 | ***0.56 (0.52-0.60)*** | 68.3 | ***0.62 (0.58-0.66)*** | 67.2 | ***0.58 (0.55-0.62)*** |  |  |
| North Africa and the Middle East | 58.6 | ***0.45 (0.44-0.46)*** | 56.1 | ***0.40 (0.38-0.42)*** | 55.6 | ***0.38 (0.36-0.40)*** | 56.9 | ***0.40 (0.37-0.42)*** | 65.3 | ***0.54 (0.51-0.57)*** |  |  |
| South-East Asia | 63.3 | ***0.48 (0.47-0.50)*** | 55.0 | ***0.35 (0.34-0.36)*** | 56.1 | ***0.36 (0.35-0.38)*** | 58.1 | ***0.40 (0.38-0.41)*** | 62.3 | ***0.45 (0.44-0.47)*** |  |  |
| North-East Asia | 57.3 | ***0.38 (0.37-0.40)*** | 56.7 | ***0.39 (0.38-0.41)*** | 58.8 | ***0.42 (0.40-0.43)*** | 57.9 | ***0.40 (0.39-0.42)*** | 61.1 | ***0.45 (0.44-0.47)*** |  |  |
| Southern and Central Asia | 42.4 | ***0.23 (0.23-0.24)*** | 41.1 | ***0.22 (0.21-0.23)*** | 42.5 | ***0.23 (0.22-0.24)*** | 43.2 | ***0.24 (0.23-0.25)*** | 51.0 | ***0.30 (0.29-0.32)*** |  |  |
| Northern America | 56.2 | ***0.39 (0.34-0.45)*** | 64.4 | ***0.53 (0.48-0.60)*** | 65.7 | ***0.56 (0.50-0.63)*** | 69.9 | ***0.67 (0.60-0.75)*** | 71.2 | ***0.67 (0.63-0.72)*** |  |  |
| South and Central America and Caribbean | 65.0 | ***0.53 (0.48-0.58)*** | 64.8 | ***0.53 (0.48-0.57)*** | 66.4 | ***0.57 (0.51-0.62)*** | 66.9 | ***0.58 (0.52-0.63)*** | 66.8 | ***0.57 (0.53-0.61)*** |  |  |
| Sub-Saharan Africa | 52.7 | ***0.35 (0.32-0.38)*** | 57.0 | ***0.40 (0.36-0.43)*** | 60.0 | ***0.43 (0.39-0.47)*** | 65.6 | ***0.53 (0.49-0.59)*** | 72.0 | ***0.70 (0.66-0.74)*** |  |  |
| p-(interaction) | **<0.001** | | | | | | | | | |  |  |

*Adjusted for maternal age at birth of infant, year of delivery of infant, pregnancy number >20weeks gestation, smoking anytime during pregnancy, remoteness of residence, area-level socio-economic status.

~Place of birth was reported by individual county or grouped according to the major categories in the Australian Bureau of Statistics Standard Australian Classification of Countries, 2011(1).

a. Oceania and Antarctica excluding Australia and New Zealand; b. North-West Europe excluding United Kingdom and Ireland.

Ɨ Pregnancy number >20 weeks gestation

ǂ Consequentially suppressed as frequencies include cells with <6.

#Codes are according to ABS Cat. No. 1270.0.55.005 – Australian Statistical Geography Standard (ASGS): Volume 5 – Remoteness Structure, 2011.(2)

≈Measured using Index of Relative Socio-economic Disadvantage, 2011.(3)

OR: odds ratio, CI: confidence interval. Italic/bold indicates significance. p-value in bold.

Table S3: Rates of participation~ (%) and adjusted odds ratios for participation in the cervical screening program in the 5-years prior to birth by place of maternal birth by different values of socio-demographic and health characteristics in women aged ≥25 years giving birth from 1 January 2000 to 30 June 2017 in New South Wales, Australia.

| Place of birth~ | % | Adjusted OR* (95%CI) | % | Adjusted OR*  (95%CI) | % | Adjusted OR*  (95%CI) | % | Adjusted OR*  (95%CI) | % | Adjusted OR*  (95%CI) |
| --- | --- | --- | --- | --- | --- | --- | --- | --- | --- | --- |
|  | **Maternal age** | | | | | | | | | |
|  | **25-29 years** | | **30-34 years** | | **35-39 years** | | **40-44 years** | | **45+ years** | |
| Australia | 76.6 | ***0.75 (0.74-0.76)*** | 83.0 | 1.00 (reference) | 84.9 | ***1.12 (1.10-1.14)*** | 84.0 | ***1.11 (1.08-1.15)*** | 82.8 | 1.10 (0.97-1.25) |
| New Zealand | 52.2 | ***0.25 (0.24-0.27)*** | 65.2 | ***0.42 (0.40-0.44)*** | 71.8 | ***0.56 (0.53-0.59)*** | 74.6 | ***0.65 (0.58-0.73)*** | 65.9 | *0.49 (0.31-0.79)* |
| Oceania and Antarctica (other)^a^ | 45.6 | ***0.17 (0.16-0.18)*** | 56.1 | ***0.26 (0.25-0.27)*** | 57.4 | ***0.29 (0.27-0.31)*** | 55.7 | ***0.31 (0.27-0.35)*** | 61.5 | *0.44 (0.28-0.70)* |
| United Kingdom and Ireland | 67.5 | ***0.46 (0.44-0.49)*** | 75.6 | ***0.62 (0.59-0.64)*** | 82.5 | ***0.85 (0.82-0.89)*** | 83.3 | ***0.92 (0.84-1.01)*** | 83.3 | 1.02 (0.70-1.49) |
| North-West Europe (other)^b^ | 58.8 | ***0.32 (0.29-0.35)*** | 70.6 | ***0.49 (0.46-0.52)*** | 77.9 | ***0.64 (0.59-0.69)*** | 78.1 | ***0.65 (0.55-0.77)*** | 76.1 | 0.74 (0.36-1.53) |
| South and Eastern Europe | 65.0 | ***0.40 (0.38-0.43)*** | 73.5 | ***0.56 (0.53-0.59)*** | 78.1 | ***0.68 (0.63-0.72)*** | 77.2 | ***0.66 (0.58-0.75)*** | 79.3 | 0.81 (0.51-1.29) |
| North Africa and the Middle East | 59.5 | ***0.30 (0.29-0.31)*** | 68.7 | ***0.45 (0.44-0.47)*** | 72.7 | ***0.59 (0.56-0.62)*** | 73.2 | ***0.67 (0.61-0.74)*** | 76.0 | 0.85 (0.59-1.22) |
| South-East Asia | 56.5 | ***0.28 (0.28-0.29)*** | 68.2 | ***0.43 (0.42-0.44)*** | 73.2 | ***0.52 (0.50-0.54)*** | 74.4 | ***0.56 (0.52-0.61)*** | 77.1 | ***0.73 (0.53-0.99)*** |
| North-East Asia | 51.4 | ***0.25 (0.24-0.26)*** | 65.7 | ***0.39 (0.38-0.40)*** | 72.8 | ***0.48 (0.46-0.49)*** | 74.1 | ***0.50 (0.47-0.54)*** | 78.2 | ***0.69 (0.48-0.98)*** |
| Southern and Central Asia | 38.8 | ***0.15 (0.15-0.15)*** | 54.6 | ***0.24 (0.23-0.25)*** | 64.2 | ***0.33 (0.31-0.34)*** | 67.0 | ***0.39 (0.34-0.44)*** | 62.7 | ***0.42 (0.24-0.72)*** |
| Northern America | 60.5 | ***0.34 (0.31-0.37)*** | 72.6 | ***0.53 (0.50-0.57)*** | 79.6 | ***0.71 (0.65-0.77)*** | 81.6 | ***0.79 (0.65-0.95)*** | 78.3 | 0.68 (0.37-1.27) |
| South and Central America and Caribbean | 65.8 | ***0.43 (0.39-0.46)*** | 72.2 | ***0.56 (0.52-0.59)*** | 79.2 | ***0.74 (0.68-0.80)*** | 81.6 | ***0.84 (0.70-1.00)*** | 75.0 | 0.62 (0.34-1.15) |
| Sub-Saharan Africa | 61.6 | ***0.34 (0.32-0.36)*** | 71.7 | ***0.47 (0.45-0.50)*** | 75.4 | ***0.55 (0.51-0.59)*** | 75.7 | ***0.60 (0.51-0.70)*** | 74.1 | 0.60 (0.34-1.08) |
| p (interaction) | **<0.001** | | | | | | | | | |
|  | **Year of delivery of infant(s)** | | | | | | | |  |  |
|  | **2000** | | **2005** | | **2010** | | **2015** | | **2017** | |
| Australia | 81.1 | 1.00 (reference) | 82.8 | ***1.07 (1.04-1.11)*** | 81.7 | 0.98 (0.94-1.01) | 78.1 | ***0.77 (0.74-0.79)*** | 76.7 | ***0.70 (0.67-0.73)*** |
| New Zealand | 65.3 | ***0.43 (0.38-0.48)*** | 64.2 | ***0.41 (0.37-0.46)*** | 65.7 | ***0.42 (0.38-0.47)*** | 63.3 | ***0.38 (0.34-0.42)*** | 57.8 | ***0.29 (0.25-0.33)*** |
| Oceania and Antarctica (other)^a^ | 50.8 | ***0.22 (0.20-0.25)*** | 56.5 | ***0.27 (0.24-0.31)*** | 54.7 | ***0.26 (0.23-0.29)*** | 49.4 | ***0.21 (0.18-0.24)*** | 50.0 | ***0.21 (0.17-0.25)*** |
| United Kingdom and Ireland | 78.0 | ***0.68 (0.62-0.75)*** | 77.3 | ***0.60 (0.55-0.67)*** | 78.4 | ***0.65 (0.59-0.72)*** | 77.5 | ***0.60 (0.55-0.67)*** | 79.0 | ***0.66 (0.57-0.76)*** |
| North-West Europe (other)^b^ | 75.2 | ***0.58 (0.48-0.70)*** | 71.1 | ***0.46 (0.38-0.54)*** | 70.0 | ***0.43 (0.37-0.50)*** | 70.0 | ***0.42 (0.36-0.49)*** | 67.4 | ***0.37 (0.31-0.45)*** |
| South and Eastern Europe | 72.9 | ***0.54 (0.47-0.61)*** | 74.0 | ***0.58 (0.51-0.66)*** | 75.3 | ***0.59 (0.52-0.67)*** | 68.7 | ***0.42 (0.37-0.46)*** | 68.4 | ***0.41 (0.35-0.48)*** |
| North Africa and the Middle East | 68.8 | ***0.49 (0.45-0.54)*** | 69.6 | ***0.48 (0.44-0.53)*** | 66.0 | ***0.42 (0.39-0.45)*** | 61.6 | ***0.34 (0.31-0.36)*** | 60.1 | ***0.30 (0.28-0.34)*** |
| South-East Asia | 68.2 | ***0.44 (0.41-0.48)*** | 67.9 | ***0.43 (0.40-0.46)*** | 65.9 | ***0.39 (0.36-0.41)*** | 63.2 | ***0.34 (0.32-0.36)*** | 61.0 | ***0.30 (0.28-0.33)*** |
| North-East Asia | 63.9 | ***0.33 (0.31-0.36)*** | 65.2 | ***0.36 (0.33-0.39)*** | 64.0 | ***0.35 (0.33-0.38)*** | 63.4 | ***0.34 (0.32-0.36)*** | 64.0 | ***0.33 (0.31-0.36)*** |
| Southern and Central Asia | 59.9 | ***0.29 (0.25-0.32)*** | 53.4 | ***0.23 (0.21-0.26)*** | 48.9 | ***0.21 (0.20-0.22)*** | 47.8 | ***0.19 (0.18-0.20)*** | 48.7 | ***0.19 (0.18-0.20)*** |
| Northern America | 75.0 | ***0.56 (0.45-0.69)*** | 73.2 | ***0.50 (0.41-0.61)*** | 74.1 | ***0.54 (0.45-0.65)*** | 71.6 | ***0.48 (0.40-0.56)*** | 71.9 | ***0.46 (0.37-0.58)*** |
| South and Central America and Caribbean | 77.1 | ***0.66 (0.54-0.81)*** | 76.8 | ***0.64 (0.53-0.78)*** | 70.7 | ***0.48 (0.41-0.56)*** | 71.7 | ***0.49 (0.42-0.57)*** | 68.8 | ***0.41 (0.34-0.50)*** |
| Sub-Saharan Africa | 71.9 | ***0.46 (0.38-0.55)*** | 73.2 | ***0.52 (0.45-0.61)*** | 71.9 | ***0.48 (0.42-0.54)*** | 69.9 | ***0.42 (0.37-0.48)*** | 65.8 | ***0.36 (0.30-0.42)*** |
| p (interaction) | **<0.001** | | | | | | | |  |  |
|  | **Pregnancy number^Ɨ^** | | | | | | | |  |  |
|  | **1** | | **2** | | **3** | | **4** | |  |  |
| Australia | 76.4 | 1.00 (reference) | 86.8 | ***2.05 (2.03-2.08)*** | 84.7 | ***1.79 (1.76-1.82)*** | 78.3 | ***1.25 (1.22-1.27)*** |  |  |
| New Zealand | 64.7 | ***0.55 (0.53-0.57)*** | 71.4 | ***0.78 (0.74-0.82)*** | 62.4 | ***0.56 (0.53-0.59)*** | 53.3 | ***0.40 (0.37-0.44)*** |  |  |
| Oceania and Antarctica (other)^a^ | 49.5 | ***0.30 (0.29-0.32)*** | 63.2 | ***0.52 (0.49-0.55)*** | 55.7 | ***0.38 (0.35-0.40)*** | 47.8 | ***0.27 (0.25-0.30)*** |  |  |
| United Kingdom and Ireland | 71.9 | ***0.66 (0.64-0.68)*** | 83.9 | ***1.31 (1.25-1.36)*** | 82.0 | ***1.14 (1.06-1.22)*** | 77.4 | ***0.87 (0.77-0.98)*** |  |  |
| North-West Europe (other)^b^ | 64.1 | ***0.48 (0.46-0.51)*** | 79.2 | 0.98 (0.91-1.05) | 77.1 | ***0.83 (0.73-0.94)*** | 73.7 | ***0.73 (0.58-0.92)*** |  |  |
| South and Eastern Europe | 64.0 | ***0.52 (0.50-0.54)*** | 79.5 | ***1.11 (1.05-1.17)*** | 81.1 | ***1.19 (1.09-1.30)*** | 78.3 | 0.98 (0.83-1.16) |  |  |
| North Africa and the Middle East | 44.9 | ***0.27 (0.26-0.28)*** | 70.9 | ***0.80 (0.77-0.83)*** | 76.4 | 1.05 (1.00-1.10) | 76.7 | 1.03 (0.97-1.10) |  |  |
| South-East Asia | 53.9 | ***0.36 (0.35-0.37)*** | 76.1 | ***0.93 (0.90-0.96)*** | 76.8 | ***0.93 (0.89-0.98)*** | 73.3 | ***0.76 (0.70-0.82)*** |  |  |
| North-East Asia | 54.4 | ***0.35 (0.34-0.36)*** | 74.4 | ***0.78 (0.76-0.80)*** | 77.9 | ***0.91 (0.86-0.97)*** | 78.3 | 0.92 (0.79-1.07) |  |  |
| Southern and Central Asia | 36.5 | ***0.20 (0.19-0.20)*** | 59.9 | ***0.46 (0.45-0.48)*** | 66.4 | ***0.59 (0.56-0.63)*** | 68.0 | ***0.62 (0.56-0.68)*** |  |  |
| Northern America | 66.8 | ***0.55 (0.51-0.58)*** | 79.7 | 1.03 (0.95-1.11) | 77.5 | 0.88 (0.77-1.00) | 72.7 | ***0.70 (0.56-0.87)*** |  |  |
| South and Central America and Caribbean | 65.6 | ***0.55 (0.52-0.58)*** | 80.8 | ***1.17 (1.09-1.26)*** | 81.3 | ***1.18 (1.04-1.34)*** | 79.4 | 1.05 (0.84-1.30) |  |  |
| Sub-Saharan Africa | 64.7 | ***0.51 (0.49-0.54)*** | 75.5 | ***0.84 (0.79-0.89)*** | 72.8 | ***0.73 (0.67-0.79)*** | 72.1 | ***0.71 (0.62-0.81)*** |  |  |
| p (interaction) | **<0.001** | | | | | | | |  |  |
|  | **Smoked anytime in pregnancy** | | | |  |  |  |  |  |  |
|  |  | **No smoking** |  | **Yes smoking** |  |  |  |  |  |  |
| Australia | 82.9 | 1.00 (reference) | 68.9 | ***0.49 (0.48-0.50)*** |  |  |  |  |  |  |
| New Zealand | 66.4 | ***0.40 (0.39-0.41)*** | 49.3 | ***0.22 (0.20-0.23)*** |  |  |  |  |  |  |
| Oceania and Antarctica (other)^a^ | 54.3 | ***0.24 (0.23-0.25)*** | 39.1 | ***0.15 (0.13-0.17)*** |  |  |  |  |  |  |
| United Kingdom and Ireland | 78.3 | ***0.65 (0.63-0.67)*** | 66.7 | ***0.38 (0.34-0.41)*** |  |  |  |  |  |  |
| North-West Europe (other)^b^ | 71.8 | ***0.48 (0.46-0.50)*** | 60.9 | ***0.31 (0.26-0.38)*** |  |  |  |  |  |  |
| South and Eastern Europe | 72.9 | ***0.54 (0.52-0.55)*** | 68.2 | ***0.43 (0.38-0.49)*** |  |  |  |  |  |  |
| North Africa and the Middle East | 66.4 | ***0.43 (0.42-0.44)*** | 64.5 | ***0.39 (0.36-0.42)*** |  |  |  |  |  |  |
| South-East Asia | 66.4 | ***0.41 (0.40-0.42)*** | 62.8 | ***0.37 (0.33-0.42)*** |  |  |  |  |  |  |
| North-East Asia | 64.2 | ***0.37 (0.37-0.38)*** | 55.3 | ***0.29 (0.24-0.34)*** |  |  |  |  |  |  |
| Southern and Central Asia | 49.4 | ***0.23 (0.22-0.23)*** | 58.3 | ***0.33 (0.26-0.42)*** |  |  |  |  |  |  |
| Northern America | 73.2 | ***0.53 (0.50-0.55)*** | 59.3 | ***0.29 (0.23-0.38)*** |  |  |  |  |  |  |
| South and Central America and Caribbean | 73.5 | ***0.57 (0.55-0.60)*** | 69.9 | ***0.50 (0.39-0.63)*** |  |  |  |  |  |  |
| Sub-Saharan Africa | 70.4 | ***0.46 (0.44-0.48)*** | 63.9 | ***0.35 (0.28-0.43)*** |  |  |  |  |  |  |
| p (interaction) | **<0.001** | | | |  |  |  |  |  |  |
|  | **Remoteness of residence**^#^ | | | | | | | |  |  |
|  | **Major city** | | **Inner regional** | | **Outer regional** | | **Remote/very remote** | |  |  |
| Australia | 82.3 | 1.00 (reference) | 81.0 | ***1.07 (1.05-1.08)*** | 79.6 | ***1.03 (1.01-1.06)*** | 72.8 | ***0.78 (0.73-0.84)*** |  |  |
| New Zealand | 64.6 | ***0.40 (0.39-0.42)*** | 69.0 | ***0.51 (0.47-0.57)*** | 62.8 | ***0.42 (0.35-0.51)*** | 61.5 | ***0.42 (0.23-0.76)*** |  |  |
| Oceania and Antarctica (other)^a^ | 53.7 | ***0.25 (0.24-0.26)*** | 58.7 | ***0.29 (0.24-0.35)*** | 42.8 | ***0.18 (0.14-0.23)*** | 38.5 | ***0.14 (0.06-0.33)*** |  |  |
| United Kingdom and Ireland | 79.1 | ***0.67 (0.66-0.69)*** | 73.9 | ***0.57 (0.52-0.62)*** | 70.5 | ***0.51 (0.43-0.61)*** | 67.7 | ***0.53 (0.30-0.95)*** |  |  |
| North-West Europe (other)^b^ | 72.8 | ***0.49 (0.47-0.52)*** | 69.5 | ***0.47 (0.41-0.54)*** | 68.6 | ***0.49 (0.38-0.64)*** | 66.7 | ***0.56 (0.17-1.84)*** |  |  |
| South and Eastern Europe | 73.6 | ***0.57 (0.55-0.59)*** | 65.3 | ***0.39 (0.34-0.46)*** | 62.2 | ***0.36 (0.25-0.51)*** | ǂ | ***0.47 (0.10-2.13)*** |  |  |
| North Africa and the Middle East | 66.7 | ***0.46 (0.45-0.47)*** | 56.8 | ***0.30 (0.25-0.35)*** | 47.6 | ***0.20 (0.13-0.29)*** | ǂ | ***0.19 (0.04-0.88)*** |  |  |
| South-East Asia | 67.5 | ***0.43 (0.43-0.44)*** | 56.3 | ***0.27 (0.25-0.30)*** | 50.5 | ***0.22 (0.19-0.26)*** | 50.0 | ***0.22 (0.12-0.40)*** |  |  |
| North-East Asia | 65.0 | ***0.38 (0.38-0.39)*** | 55.9 | ***0.27 (0.24-0.30)*** | 50.0 | ***0.22 (0.17-0.28)*** | 57.1 | ***0.32 (0.14-0.74)*** |  |  |
| Southern and Central Asia | 50.6 | ***0.24 (0.23-0.24)*** | 40.9 | ***0.17 (0.15-0.19)*** | 30.0 | ***0.10 (0.08-0.12)*** | 28.3 | ***0.11 (0.06-0.20)*** |  |  |
| Northern America | 74.7 | ***0.55 (0.52-0.58)*** | 69.0 | ***0.47 (0.41-0.54)*** | 69.6 | ***0.48 (0.37-0.64)*** | 57.9 | ***0.28 (0.10-0.79)*** |  |  |
| South and Central America and Caribbean | 75.0 | ***0.60 (0.57-0.63)*** | 65.3 | ***0.40 (0.32-0.49)*** | 56.8 | ***0.28 (0.17-0.46)*** | ǂ | 0.27 (0.06-1.12) |  |  |
| Sub-Saharan Africa | 71.5 | ***0.48 (0.46-0.50)*** | 65.0 | ***0.39 (0.34-0.45)*** | 65.4 | ***0.40 (0.29-0.56)*** | 45.0 | 0.15 (0.06-0.39) |  |  |
| p (interaction) | **<0.001** | | | | | | | |  |  |
|  | **Area-level socio-economic status (Quintile)** ≈ | | | | | | | | | |
|  | **1 (most disadvantaged)** | | **2** | | **3** | | **4** | | **5 (least disadvantaged)** | |
| Australia | 76.7 | ***0.68 (0.67-0.69)*** | 80.1 | ***0.78 (0.77-0.80)*** | 82.5 | ***0.88 (0.87-0.90)*** | 83.9 | ***0.95 (0.93-0.97)*** | 85.6 | 1.00 (reference) |
| New Zealand | 50.7 | ***0.22 (0.21-0.23)*** | 59.3 | ***0.29 (0.27-0.31)*** | 64.4 | ***0.34 (0.32-0.37)*** | 70.0 | ***0.42 (0.39-0.46)*** | 76.5 | ***0.55 (0.52-0.58)*** |
| Oceania and Antarctica (other)^a^ | 45.8 | ***0.15 (0.14-0.16)*** | 52.5 | ***0.19 (0.18-0.20)*** | 55.8 | ***0.21 (0.19-0.23)*** | 62.7 | ***0.28 (0.25-0.31)*** | 71.9 | ***0.41 (0.36-0.45)*** |
| United Kingdom and Ireland | 73.1 | ***0.47 (0.43-0.52)*** | 75.2 | ***0.52 (0.48-0.55)*** | 78.8 | ***0.61 (0.57-0.65)*** | 79.6 | ***0.64 (0.60-0.68)*** | 79.5 | ***0.62 (0.60-0.64)*** |
| North-West Europe (other)^b^ | 68.8 | ***0.38 (0.33-0.44)*** | 72.2 | ***0.46 (0.41-0.52)*** | 72.4 | ***0.45 (0.40-0.50)*** | 73.0 | ***0.48 (0.43-0.53)*** | 72.6 | ***0.44 (0.41-0.46)*** |
| South and Eastern Europe | 71.2 | ***0.44 (0.40-0.47)*** | 71.3 | ***0.44 (0.41-0.48)*** | 73.3 | ***0.48 (0.44-0.51)*** | 75.8 | ***0.54 (0.50-0.58)*** | 74.1 | ***0.49 (0.46-0.52)*** |
| North Africa and the Middle East | 66.8 | ***0.36 (0.35-0.37)*** | 64.5 | ***0.32 (0.30-0.34)*** | 63.4 | ***0.30 (0.28-0.32)*** | 65.9 | ***0.33 (0.31-0.36)*** | 72.5 | ***0.44 (0.41-0.48)*** |
| South-East Asia | 69.9 | ***0.39 (0.37-0.40)*** | 62.9 | ***0.29 (0.28-0.30)*** | 63.8 | ***0.30 (0.28-0.31)*** | 64.9 | ***0.32 (0.30-0.33)*** | 69.8 | ***0.38 (0.36-0.40)*** |
| North-East Asia | 62.7 | ***0.29 (0.28-0.30)*** | 62.5 | ***0.31 (0.29-0.32)*** | 64.7 | ***0.33 (0.31-0.34)*** | 63.9 | ***0.32 (0.31-0.33)*** | 68.1 | ***0.37 (0.36-0.39)*** |
| Southern and Central Asia | 48.9 | ***0.18 (0.17-0.18)*** | 47.6 | ***0.17 (0.17-0.18)*** | 49.0 | ***0.18 (0.17-0.19)*** | 49.6 | ***0.19 (0.18-0.20)*** | 59.9 | ***0.26 (0.25-0.27)*** |
| Northern America | 65.0 | ***0.33 (0.28-0.38)*** | 70.5 | ***0.42 (0.38-0.48)*** | 71.2 | ***0.43 (0.38-0.49)*** | 75.8 | ***0.54 (0.48-0.62)*** | 76.5 | ***0.53 (0.50-0.57)*** |
| South and Central America and Caribbean | 74.5 | ***0.50 (0.45-0.56)*** | 74.6 | ***0.50 (0.46-0.55)*** | 74.8 | ***0.51 (0.46-0.57)*** | 75.4 | ***0.53 (0.48-0.58)*** | 73.6 | ***0.48 (0.44-0.52)*** |
| Sub-Saharan Africa | 61.5 | ***0.28 (0.26-0.31)*** | 65.0 | ***0.32 (0.29-0.35)*** | 69.2 | ***0.37 (0.34-0.42)*** | 73.0 | ***0.45 (0.40-0.49)*** | 77.8 | ***0.56 (0.53-0.60)*** |
| p (interaction) | **p<0.001** | | | | | | | | | |

*Adjusted for maternal age at birth of infant, year of delivery of infant, pregnancy number >20weeks gestation, smoking anytime during pregnancy, remoteness of residence, area-level socio-economic status.

~Place of birth was reported by individual county or grouped according to the major categories in the Australian Bureau of Statistics Standard Australian Classification of Countries, 2011^1^.

a. Oceania and Antarctica excluding Australia and New Zealand; b. North-West Europe excluding United Kingdom and Ireland.

Ɨ Pregnancy number >20 weeks gestation.

ǂ Consequentially suppressed as frequencies include cells with <6.

#Codes are according to ABS Cat. No. 1270.0.55.005 – Australian Statistical Geography Standard (ASGS): Volume 5 – Remoteness Structure, 2011.^3^

≈Measured using Index of Relative Socio-economic Disadvantage, 2011.^4^

OR: odds ratio, CI: confidence interval. Italic/bold indicates significance. p-value in bold.

**References:**

1. Australian Bureau of Statistics (ABS). ABS Cat. No. 1269.0 - Standard Australian Classification of Countries (SACC), 2011. 2011. <https://www.abs.gov.au/AUSSTATS/abs@.nsf/allprimarymainfeatures/F63BD8670E4CF23CCA257FDC0011DB93?opendocument=> (accessed 8 February 2022).

2. Australian Bureau of Statistics (ABS). Reflecting a nation: Stories from the 2011 census, 2012–2013. 2012. <https://www.abs.gov.au/ausstats/abs@.nsf/lookup/2071.0main+features902012-2013> (accessed 6 December 2022)

3. Australian Bureau of Statistics (ABS). 1270.0.55.005 - Australian Statistical Geography Standard (ASGS): Volume 5 - Remoteness Structure, July 2011. 2011. <https://www.abs.gov.au/AUSSTATS/abs@.nsf/Lookup/1270.0.55.005Explanatory%20Notes10July%202011?OpenDocument> (accessed 8 February 2022).

4. Australian Bureau of Statistics (ABS). 2033.0.55.001 - Socio-economic Indexes for Areas (SEIFA), Data Cube only, 2011. Table 2. Statistical Area Level 2 (SA2) Index of Relative Socio-economic Disadvantage. 2011. <https://www.abs.gov.au/AUSSTATS/abs@.nsf/second+level+view?ReadForm&prodno=2033.0.55.001&viewtitle=Census%20of%20Population%20and%20Housing:%20Socio-Economic%20Indexes%20for%20Areas%20(SEIFA),%20Australia~2011~Previous~28/03/2013&&tabname=Past%20Future%20Issues&prodno=2033.0.55.001&issue=2011&num=&view=&>. (accessed 16 February 2022).
